# On Entropy of Probability Integral Transformed Time Series

**DOI:** 10.3390/e22101146

**Published:** 2020-10-12

**Authors:** Dragana Bajić, Nataša Mišić, Tamara Škorić, Nina Japundžić-Žigon, Miloš Milovanović

**Affiliations:** 1 Faculty of Technical Sciences, University of Novi Sad, 21000 Novi Sad, Serbia; tamara.ceranic@gmail.com; 2 Research and Development Institute Lola Ltd., 11000 Belgrade, Serbia; natasa.misic@li.rs; 3 Faculty of Medicine, University of Belgrade, 11000 Belgrade, Serbia; nzigon@med.bg.ac.rs; 4 Mathematical Institute of the Serbian Academy of Sciences and Arts, 11000 Beograd, Serbia; milosm@mi.sanu.ac.rs

**Keywords:** approximate and sample entropy, cross-entropy, copulas, probability integral transformation, dependency structures

## Abstract

The goal of this paper is to investigate the changes of entropy estimates when the amplitude distribution of the time series is equalized using the probability integral transformation. The data we analyzed were with known properties—pseudo-random signals with known distributions, mutually coupled using statistical or deterministic methods that include generators of statistically dependent distributions, linear and non-linear transforms, and deterministic chaos. The signal pairs were coupled using a correlation coefficient ranging from zero to one. The dependence of the signal samples is achieved by moving average filter and non-linear equations. The applied coupling methods are checked using statistical tests for correlation. The changes in signal regularity are checked by a multifractal spectrum. The probability integral transformation is then applied to cardiovascular time series—systolic blood pressure and pulse interval—acquired from the laboratory animals and represented the results of entropy estimations. We derived an expression for the reference value of entropy in the probability integral transformed signals. We also experimentally evaluated the reliability of entropy estimates concerning the matching probabilities.

## 1. Introduction

The sampling theorem [1] paved a way for pervasive signal processing within the scientific fields where it was once inconceivable. Tools developed for classical thermodynamics or communications engineering found new multidisciplinary implementation. 

The function developed to estimate the uncertainty of the communication signals—entropy [2]—attracted the attention of scientists from a range of different fields. Other entropy concepts were accepted as well—Kolmogorov–Sinai [3], Grassberger et al. [4] and Eckmann et al. [5], despite difficult implementation and firm theoretical framework. 

To ensure easy implementation, Pincus [6] proposed the approximate entropy (*ApEn*) that avoids the rigid mathematical requirements of its theoretical predecessors (hence the name—approximate). The researchers readily accepted *ApEn* and its modification *SampEn* (sample entropy, [7]), with a commendation of the rapidly growing number of citations [8]. 

Medical researchers quickly realized the benefits of signal processing [9] and successfully applied *ApEn* and *SampEn*, in particular for cardiovascular signals: for heart rate variability (HRV) analysis in patients with type 2 diabetes [10], in patients with heart failure [11], in healthy subjects [12] during exercise and during resting [13], under stressful conditions [7,14] or for age and gender analysis [15,16]. Entropy became important for quantifying the deterministic chaos of HRV [17], cardiac variability [18], and complexity changes in cardiovascular disease [19], but it can also be applied for monitoring the changes in neurocardiovascular dynamics, e.g., in acute brain injury patients [20].

Cross-entropy (*XEn*) was a straightforward generalization, derived as a measure that estimates the mutual (un)predictability of two simultaneously recorded and interconnected signals [7,21,22]. *XEn* can be based on *ApEn* (*XApEn*) or *SampEn* (*XSampEn*). It came into the focus slower than *ApEn* and *SampEn,* but it has found its place in cardiovascular analytics [23,24].

Many contributions have introduced the improvement of the original concepts [14,25,26,27,28] or proposed the alternative ways to approximate the entropy [8,29,30,31,32], but *ApEn* and *SampEn* have remained on the top, with the advantages summarized in a comprehensive tutorial [33].

The entropy application proposed in this paper is motivated by a mathematical method based on copula theory [34]. The approach decomposes a multivariate joint distribution of *D* > 1 signals, each with an arbitrary distribution, into *D* independent uniform marginals and a function that binds them all—the copulas. The copulas reveal the dependency structure of two or more related signals, while independent marginals, with equalized amplitude distributions, preserve the shape of signal fluctuations. The signal transformation that produces the copulas and the corresponding marginals is called the probability integral transform (PIT, or PI-transform) [35].

The goal of this paper is to investigate the changes of entropy when the amplitude distribution of the time series is equalized, while the temporal fluctuations of the signal amplitudes remain intact. In particular, we aim to show the benefits of the probability integral transformation to the reliability of entropy estimates. We used artificial time series—pseudo-random signals with arbitrary distributions, coupled with statistical or deterministic methods that include generators of statistically dependent distributions, linear and non-linear transforms, and unpredictable (but not random) signals of deterministic chaos. We also used real cardiovascular time series—systolic blood pressure and pulse interval—acquired from laboratory animals.

The paper is organized as follows: Section 2 explains Sklar’s theorem, copula distribution, and probability integral transform that motivated this study. It also gives a brief recap of *ApEn, SampEn,* and *XEn* procedures. Section 2 then continues by presenting the artificial signals that were used to verify the proposed concept, including pseudo-random signals with Gaussian, beta, and gamma distributions and deterministic chaos (logistic map [36]). The pairs of signals are mutually coupled with correlation coefficient ranging from zero to one. The dependency of the signal samples is achieved by moving average (MA) filter, or by non-linear equations. The applied coupling methods are checked using statistical tests for correlation and autocorrelation. The changes in signal regularity are verified by a multifractal spectrum. The section concludes with a brief description of the experimental protocol and of the signal acquisition from the laboratory animals. Section 3 first empirically defines the threshold, a parameter crucial for reliable entropy estimation. The results of entropy estimation from the artificial signals and the signals of laboratory animals exposed to stress are presented and discussed. An expression for the entropy of random signals with uniform distribution is derived, a reference value to which the entropy of PI-transformed signals can be compared. This expression can be applied for *ApEn, SampEn,* and *XEn.* The reliability of entropy estimates concerning the matching probabilities is also experimentally evaluated. Concluding remarks are given in Section 4.

## 2. Materials and Methods

This section shows the theory underlying the dependency structures of multivariate time series given by a copula density. The copula density was a motivation to estimate the entropy from signals with equalized amplitudes. The *ApEn, SampEn*, and *XEn* procedures are also briefly described. The signals to be analyzed are the cardiovascular time series recorded from rats exposed to various stressful situations. However, since the PI-transform influence to entropy estimates are still unknown, first we need to analyze the artificially generated signals with known statistical properties.

### 2.1. Probability Integral Transform, Sklar’s Theorem and Copula Density

**Figure 1:**
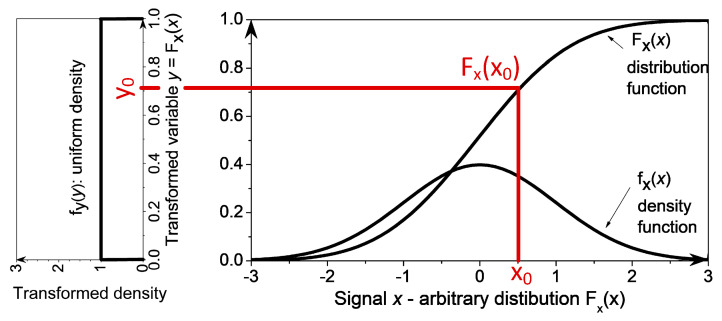
Probability integral transform. The random variable *x* with the density function *f_x_*(*x*) is transformed into the uniformly distributed random variable *y* = *F_x_*(*x*), with uniform density *f_y_*(*y*) = 1.

The probability integral transform (PIT, or PI-transform) converts a random variable (RV) *x* with an arbitrary distribution function *F_x_*(*x*) into a RV *y* uniformly distributed on the segment [0, 1] [35]. The function used for transformation is the distribution function of signal *x*, i.e., *y* = *F_x_*(*x*). The resulting distribution function *F_y_*(*y*) is uniform (Figure 1). The proof can be found in textbooks on probability and random variables (e.g., p. 139, [37]), but it is included for the completeness:

From Figure 1 it is obvious that the probabilities Pr{*x* ≤ *x*_0_} and Pr{*y* ≤ *y*_0_} are equal. The same applies to the distribution functions: (*F_x_*(*x*_0_) = Pr{*x* ≤ *x*_0_}) = (*F_y_*(*y*_0_) = Pr{*y* ≤ *y*_0_}). Additionally, the PIT transformation rule states that *y*_0_ = *F_x_*(*x*_0_), so the following may be written:

(1) $$F_{y}\left(y_{0}\right)=\mathrm{Pr}\left\{y\leq y_{0}\right\}=\mathrm{Pr}\left\{x\leq x_{0}\right\}=\mathrm{Pr}\left\{x\leq F_{x}^{-1}\left(y_{0}\right)\right\}=F_{x}\left(F_{x}^{-1}\left(y_{0}\right)\right)=y_{0}$$

The distribution function F*_y_*(*y*_0_) is a linear function of *y*_0_. Its derivative is a constant, so the distribution of signal *y* is indeed uniform.

**Figure 2:**
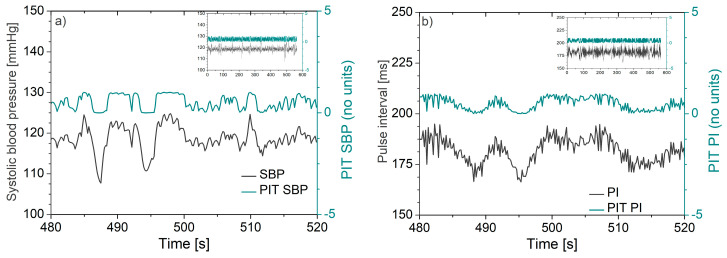
Waveforms recorded from a laboratory rat before and after the probability integral transform: (**a**) Systolic blood pressure (SBP) and corresponding probability integral transform (PIT) signal and (**b**) pulse interval (PI) and corresponding PIT signal. Note that fluctuations of PIT signals follow the fluctuations of the original signals. The insets in the top right corner illustrate the uniformity of the PIT amplitudes along the entire recording time.

An illustrative example of signals transformed by PIT is presented in Figure 2, showing the systolic blood pressure (SBP) and pulse interval (PI) of a laboratory rat before and after PIT application.

PIT gained its popularity during the early days of the digital era, as its inverse produces a random signal of arbitrary distribution. When software packages started to provide built-in distribution generators, PIT was almost let into oblivion, but not for long. Sklar’s theorem [38], although derived in the early sixties, came into the research focus at the turn of the century and brought PIT to the forefront again.

Sklar’s theorem states that every *D*-dimensional (multivariate) distribution function $H\left(x_{01},x_{02},\ldots ,x_{0D}\right)=\mathrm{Pr}\left\{x_{1}\leq x_{01},\ldots ,x_{D}\leq x_{0D}\right\}$ can be expressed in terms of its uniform marginals $F_{xi}\left(x_{0i}\right)=\mathrm{Pr}\left\{x_{i}\leq x_{0i}\right\},\;i=1,\ldots ,D,$ and a joint distribution—a copula *C*—that binds them, i.e.,

(2) $$H\left(x_{01},x_{02},\ldots ,x_{0D}\right)=C\left(F_{x1}\left(x_{01}\right),F_{x2}\left(x_{02}\right),\ldots ,F_{xD}\left(x_{0D}\right)\right).$$

An alternative interpretation can be formulated if we recollect that each marginal is uniformly distributed, i.e., $F_{xi}\left(x_{0i}\right)=u_{i},\;i=1,\ldots ,D$:

(3) $$C\left(u_{1},u_{2},\ldots ,u_{D}\right)=\mathrm{Pr}\left\{x_{1}\leq F_{x1}^{-1}\left(u_{1}\right),x_{2}\leq F_{x2}^{-1}\left(u_{2}\right),\ldots x_{D}\leq F_{xD}^{-1}\left(u_{D}\right)\right\}.$$

Despite the abstract theoretical definition, a copula implementation and interpretation are simple. The copulas are distribution functions, and their derivatives are the probability density functions—the copula density.

The copula density depicts the dependency structure (density of dependency) of the composite signals. An ability to visualize the dependency structure, especially for bivariate signals, is a unique advantage of the copulas density. To estimate the empirical copula density—it is sufficient to apply the probability integral transform to the source signals and find their joint probability density function.

**Figure 3:**
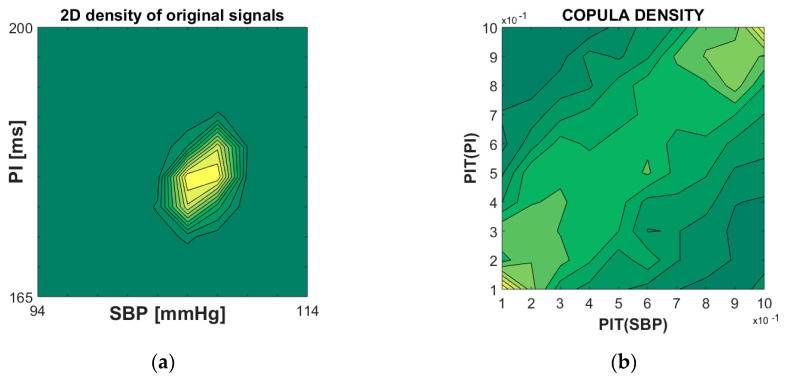
(**a**) Joint probability density function of systolic blood pressure (SBP) and pulse interval (PI) of a laboratory rat; (**b**) copula density of PI-transformed SBP and PI signals. Note that the linear dependency structure along the diagonal in (**b**) is consistent with the linear cardiovascular relationship of SBP and PI in healthy subjects.

An example of empirical copula density is shown in Figure 3. The left panel shows the classical joint probability distribution function of systolic blood pressure (SBP) and pulse intervals (PI) signals of the laboratory rats. The right panel presents the copula density of the same signals (*D* = 2), in the abstract two-dimensional [0, 1]*^D^* copula plane. The dependency structure reveals the linear relationship between SBP and PI that corresponds to baroreflex, a major regulatory feedback that helps to maintain blood pressure at a nearly constant level.

Another property of the copulas is that they quantify the strength of signal coupling. It differs from other similar procedures because it can handle more than two signals, it can capture non-linear dependencies as well, but, above all, it can be adapted to the properties of the observed signals. There are many copula sets (“copula families” [39]), and each one is adapted to the particular signal type. For example, Frank copulas are the most suitable for cardiovascular signals [34]. It is this feature of the copulas that has brought them great popularity in many research domains, from finances [40], telecommunications [41], civil engineering [42], geodesy, [43] climatology [44], to medicine [45] and cardiology [34].

### 2.2. XEn, ApEn and SampEn

The procedures for estimating *ApEn* and *SampEn*, originally introduced in [6,7], are repeatedly described in most papers that implement them. We shall give a brief recap of *XEn* as a general procedure and outline the differences in respect to *ApEn* and *SampEn*.

*XEn* [25,46] measures the mutual (un)predictability of time series ***X*** and ***Y***, each one comprising *N* samples:

- Reference series *x_i_* ∈ ***X***, *i* = 1, …, *N*;
- Follower series *y_j_* ∈ ***Y***, *j* = 1, …, *N*.

If A*pEn* or *SampEn* are implemented, there is just a single series ***X*** and in the remaining explanation ***Y*** = ***X***.

Time series must be pre-processed before further analysis. The signal comparability is ensured by z-normalization (standard scaling—mean signal value and standard deviation reduced to 0 and 1, respectively). The estimation of statistical moments requires the stationary time series, ensured by a filter designed specifically for biomedical time series [47].

Time series are then divided into the overlapping vectors of length *m* (*m* is usually 2, 3 or 4):

- Template vector $X_{m}^{\left(i\right)}=\left[x_{i},x_{i+1},\cdots ,x_{i+m-1}\right],\;i=1,\cdots ,N-m+1$;
- Follower vector $Y_{m}^{\left(j\right)}=\left[y_{j},y_{j+1},\cdots ,y_{j+m-1}\right],\;j=1,\cdots ,N-m+1$;

A distance between each template $X_{m}^{\left(i\right)}$ and each follower vector $Y_{m}^{\left(j\right)}$ is defined as a maximal absolute sample difference:

- $d\left(X_{m}^{\left(i\right)},Y_{m}^{\left(j\right)}\right)=\underset{k=0,\cdots ,m-1}{max}\left|x_{i+k}\;-\;y_{j+k}\right|,\;i,j=1,\cdots ,N-m+1$.

If the distance is less than or equal to the predefined threshold *r*, the vectors are declared as similar. The template matching probability ${\hat{p}}_{i}^{\left(m\right)}\left(r\right)$ is proportional to the number of vectors similar to a particular template vector $X_{m}^{\left(i\right)}$:

(4) $${\hat{p}}_{i}^{\left(m\right)}\left(r\right)=\mathrm{Pr}\left\{d\left(X_{m},Y_{m}\right)\leq r|X_{m}=X_{m}^{\left(i\right)},\;Y_{m}\in Y,\;r>0\right\}\;=\frac{1}{N-m+1}\cdot \overset{N-m+1}{\underset{j=1}{\sum }}I\;\{d\left(X_{m}^{\left(i\right)},X_{m}^{\left(i\right)}\right)\leq r\}.$$

The sign “^” in (4) denotes an estimate, while *I*{·} is an indicator function that is equal to 1 if $d\left(X_{m}^{\left(i\right)},Y_{m}^{\left(j\right)}\right)\leq r$, otherwise it is equal to zero. It is used as a mathematical description for the counting process, so (4) estimates the relative frequency of vectors similar to the template $X_{m}^{\left(i\right)}$. For *SampEn* and *XSampEn*, the step (*N* − *m* + 1) is excluded from averaging. *SampEn* also excluded self-matching, when the template vector $X_{m}^{\left(i\right)}$ is compared to itself.

Averaging the probabilities is different for *ApEn* and *SampEn*, and the corresponding cross-entropies. For (*X*) *ApEn*, the logarithms of the probabilities (the information contents of each template [2]) are averaged:

(5) $${\hat{\Phi }}^{\left(m\right)}\left(r,N\right)=\frac{1}{N-m+1}\cdot \overset{N-m+1}{\underset{i=1}{\sum }}\ln({\hat{p}}_{i}^{\left(m\right)}\left(r\right)).$$

For *SampEn*, the logarithm is taken from the averaged probabilities:

(6) $${\hat{\Psi }}^{\left(m\right)}\left(r,N\right)=\ln\left(\frac{1}{N-m}\cdot \overset{N-m}{\underset{i=1}{\sum }}{\hat{p}}_{i}^{\left(m\right)}\left(r\right)\right).$$

The complete procedure is repeated for the vectors of length *m* + 1, with summands equal to:

(7) $${\hat{\Phi }}^{\left(m+1\right)}\left(r,N\right)=\frac{1}{N-m}\cdot \overset{N-m}{\underset{i=1}{\sum }}\ln({\hat{p}}_{i}^{\left(m+1\right)}\left(r\right)),\;{\hat{\Psi }}^{\left(m+1\right)}\left(r,N\right)=\ln\left(\frac{1}{N-m}\cdot \overset{N-m}{\underset{i=1}{\sum }}{\hat{p}}_{i}^{\left(m+1\right)}\left(r\right)\right)$$

Final entropy estimates are:

(8) $$\hat{\left(X\right)ApEn}\left(m,r,N\right)={\hat{\Phi }}_{}^{\left(m\right)}\left(r,N\right)-{\hat{\Phi }}_{}^{\left(m+1\right)}\left(r,N\right),\;\hat{\left(X\right)SampEn}\left(m,r,N\right)={\hat{\Psi }}_{}^{\left(m\right)}\left(r,N\right)-{\hat{\Psi }}_{}^{\left(m+1\right)}\left(r,N\right)=\ln\left(\frac{\sum_{i=1}^{N-m}{\hat{p}}_{i}^{\left(m\right)}\left(r\right)}{\sum_{i=1}^{N-m}{\hat{p}}_{i}^{\left(m+1\right)}\left(r\right)}\right)$$

While the sample entropy is a robust estimator [7], the approximate entropy can suffer from inconsistencies as it is based on the logarithm of probability estimations with accumulating estimation errors (cf. Equation (5)). The time-series length *N* and threshold *r* are both pointed out as the primary cause for inconsistencies [7,14,22,25]. One of the main adverse outcomes is the zero matching probability (a template vector with no similar followers). The various corrections proposed in [8,22] converge towards the true entropy values when time series length *N* converges to infinity. We implemented a simple correction, which turned out to be close to the true entropy value regardless of *N* [22]:

(9) $${\hat{\Phi }}_{C}^{\left(m\right)}\left(r,N\right)=\frac{1}{N-m+1-N_{0}}\cdot \overset{N-m+1}{\underset{i=1,\;{\hat{p}}_{i}^{\left(m\right)}\left(r\right)\neq 0}{\sum }}\ln\left({\hat{p}}_{i}^{\left(m\right)}\left(r\right)\right),\;N_{0}=\overset{N-m+1}{\underset{j=1}{\sum }}I\;\{{\hat{p}}_{i}^{\left(m\right)}\left(r\right)=0\}.$$

As already mentioned, this paper aims to investigate the *ApEn*, *SampEn,* and *XEn* of PI-transformed cardiovascular time series, and PIT is closely related to the copula density. However, another entropy measure related to the copulas—the copula entropy—already exists [48,49,50]. It is based on the Shannon entropy [2], with corresponding probabilities evaluated as normalized histograms of the empirical copula density.

The Shannon entropy applied to a time series is a static measure. If the order of signal samples is permuted in time, as in isodistributional surrogate signals [51], the Shannon entropy will remain unaltered because the probability density function remains the same, as it is derived from the amplitude values, regardless of their position in time. Hence, Shannon entropy reflects the level of orderliness in spatial, but not in the temporal domain.

The *ApEn*-based entropies reveal the signal orderliness both in the spatial domain (via threshold *r*) and in the temporal domain (via the vector length *m* and its increase to *m* + 1). However, if the threshold *r* is set to zero, it was shown that *ApEn* is equivalent to the Shannon conditional entropy applied to the first-order Markov chain [6]. This is, however, a theoretical abstraction, as *ApEn* with *r* = 0 cannot be practically achieved.

It was also shown that *ApEn* of differentially coded binary time series for *r* = 0 is equivalent to Shannon’s binary entropy [52]. This result, although feasible, has no practical application.

### 2.3. Artificial Time Series

To test the entropy of signals before and after the PI-transformation, we generated random signals with Gaussian, gamma and beta distribution. Gamma and beta distributions, with parameters (α, β) equal respectively to (1, 2) and (3, 1), are skewed distributions with amplitude concentrated in different regions. For each example, we have generated 20 signals or signal pairs, comprising *N* = 3000 samples each, and the distribution of each signal was tested by the Kolmogorov–Smirnov test. Additionally, we generated signals of deterministic chaos, where their unpredictability is governed by deterministic laws of the simple non-linear equation of the logistic map [36]:

(10) x(i+1)=RP·x(i)·(1−x(i))

**Figure 4:**
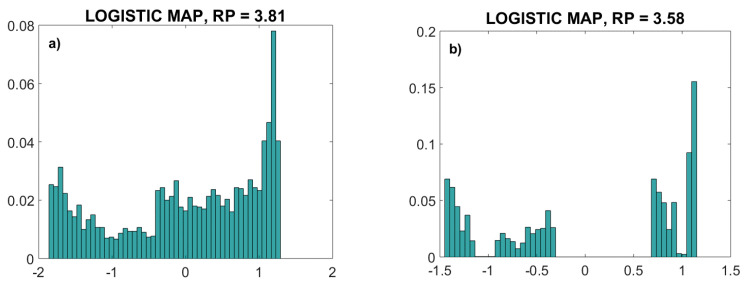
Normalized histogram of logistic map deterministic chaos time series. (**a**): parameter *RP* = 3.81. (**b**): parameter *RP* = 3.58. The signal in (**b**) is not continuous, so it does not fulfill the theoretical requirements for copula estimation.

The parameter *RP* was chosen to be 3.81, a value that guarantees chaotic behavior over the complete signal range without oscillations. Another value, *RP* = 3.58, generated a chaotic signal, but omitting some amplitude ranges. The probability distribution function (a normalized histogram) of these two signals is presented in Figure 4. The second signal (*RP* = 3.58) is an example of the signal that cannot be used for the copulas as it is not continuous, so it does not fulfill the theoretical requirements.

The statistical dependency of signal samples is induced using an MA filter:

(11) $$x^{\left(k\right)}\left(i\right)=\frac{1}{k}\cdot \overset{k-1}{\underset{j=0}{\sum }}x\left(i+j\right),\;i=1,\ldots ,N-k+1,\;k=2,\ldots ,10$$

Statistically dependent time series with distribution functions $F_{x}\left(x\right)$ and $F_{y}\left(y\right)$ were created using the copula method as follows: the original signal points (*x*, *y*) are PI-transformed into uniform signal points (*u_x_*, *u_y_*) and then the corresponding joint distribution is created in the unit plane [0, 1]^2^ using the Frank copula distribution [34,39]:

(12) $$C\left(u_{x},u_{y}\right)=-\frac{1}{\theta }\cdot \log\left\{1+\frac{\left(e^{-\theta \cdot u_{x}}-1\right)\cdot \left(e^{-\theta \cdot u_{y}}-1\right)}{e^{-\theta }-1}\right\}$$

Finally, each $\left(u_{x},u_{y}\right)$ point is transformed back to the original signal plane using the transform $\left(x,y\right)=\left(F_{x}^{-1}\left(u_{x}\right),\;F_{y}^{-1}\left(u_{y}\right)\right).$ This method generates mutually dependent time series ***X*** and ***Y*** with distributions $F_{x}\left(x\right)$ and $F_{y}\left(y\right)$, where the dependency level is given by the copula parameter *θ*.

**Figure 5:**
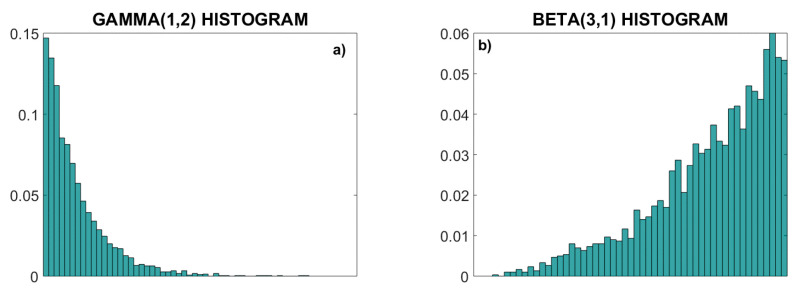

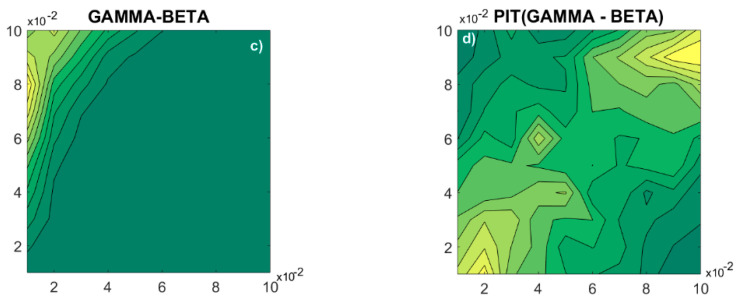
Statistical properties of skewed distributions. (**a**) Normalized histogram (empirical PDF) of the gamma (1, 2) distribution; (**b**) normalized histogram of the beta (3, 1) distribution; (**c**) joint 2D PDF; (**d**) copula density. Note that the linear statistical dependency induced by the copula parameter θ=3 cannot be recognized in the joint probability density function (**c**), but it is visible in the dependency structure of the copula density (**d**).

Figure 5 presents an illustrative example of signals ***X*** and ***Y*** with skewed gamma and beta distributions, generated using copula generator with parameter *θ* = 3. From their joint probability density function (PDF) (Figure 5c) no conclusions can be drawn about the relationship of ***X*** and ***Y***, but strong linear coupling is clearly visible in their dependency structure (empirical copula density, Figure 5d).

Deterministic non-linear dependency is introduced using relationships ***Y*** = *a*·***X****^EXP^* + *b*, where *a* = *b* = 1 are arbitrary chosen parameters, while parameter *EXP* ranges from 1.1 to 2.

**Figure 6:**
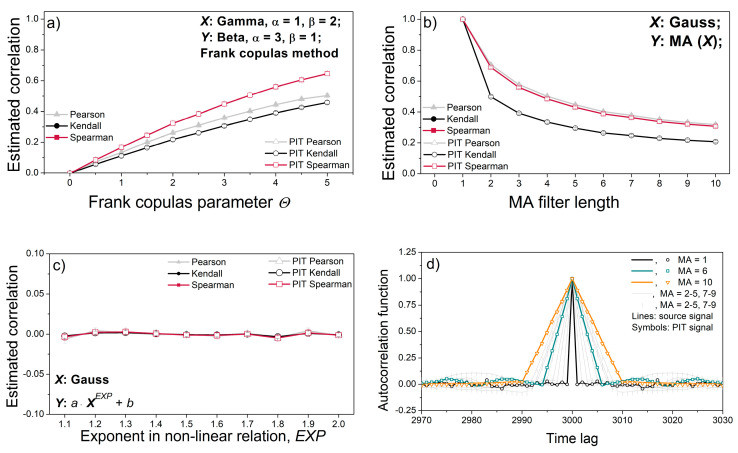
Classical properties of source signals and PI-transformed signals. (**a**) Correlation coefficients of the gamma–beta dependent signal pairs; (**b**) correlation coefficient of Gaussian signal pairs with dependency induced by the moving average (MA) filter; (**c**) correlation coefficient of non-linear dependencies; (**d**) autocorrelation function of a single signal filtered by the MA filter. The results are presented as the mean ± SE (standard error of mean) of 20 signals or signal pairs.

Figure 6a,b show the linear signal coupling estimated by the Pearson, Kendall and Spearman tests [53]. All three tests use different procedures—the Pearson test uses classical moment theory, while the Kendall and Spearman tests use different ranking procedures. The aim was to examine whether the PI transformation changes the linear coupling of two data series. The corresponding data series are generated using a copula generator and an MA filter.

Figure 6a,b show that the PI transformation does not cause changes in the linear dependence of the two signals. The dependence between adjacent samples of a single signal also remains unchanged after PI-transformation, as shown by the autocorrelation function in Figure 6d. However, the Pearson, Spearman, and Kendall tests, designed to capture correlation, are unable to capture nonlinear dependence, Figure 6c.

The correlation lines of source signals and their PI-transformed counterparts in Figure 6 overlap perfectly, showing that PIT does not alter the coupling of the signal pairs.

To test whether the differentiation would be possible at all, we estimated a multifractal spectrum that describes the fluctuation of the local regularity of the observed signals. The multifractal spectrum is estimated in terms of wavelet leaders [54].

**Figure 7:**
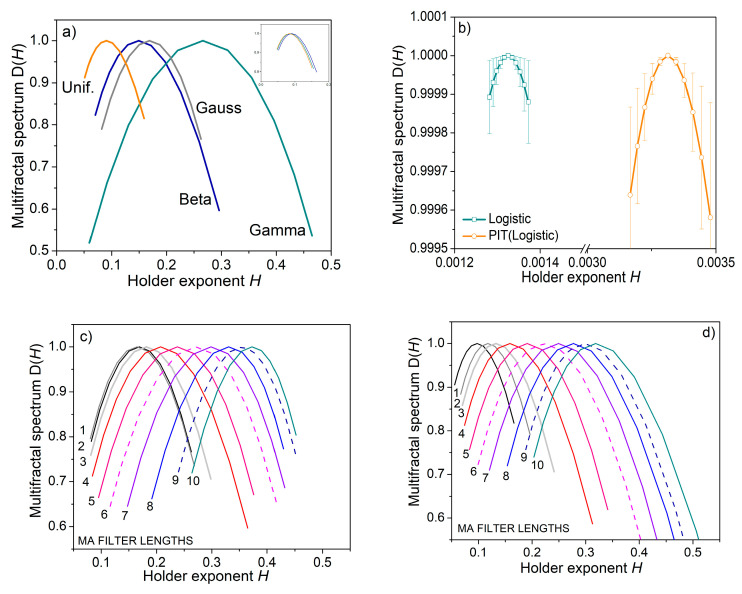
Multifractal spectrum of artificial test signals. (**a**) Spectrum of pseudorandom signals with different distributions; inset in the top right corner shows the spectrum of PI transformed signals; (**b**) spectrum of logistic map signal (deterministic chaos); (**c**) spectra of the same signal submitted to the MA filter; (**d**) spectra of PI-transformed signals from (**c**).

The results in Figure 7 reveal that the PIT induced the changes in signal regularity. The inset in the upper right corner of Figure 7a shows that the spectra of the transformed pseudorandom signals differ from the original spectra; besides, all transformed spectra overlap as the signals get the same distribution (inset in Figure 7a). The spectrum of deterministic chaos reveals monofractal properties. The spectrum remains monofractal after PI-transform (Figure 7b). The spectra of signals filtered by the MA filter differ from the source signals, showing that the local regularity of the signals has changed.

### 2.4. Time Series Recorded from the Laboratory Rats Exposed to Shaker and Restraint Stress

The real cardiovascular signals we used to check the PIT entropy concept were recorded at Laboratory of Cardiovascular Pharmacology, Medical Faculty, University of Belgrade, from outbred male Wistar rats weighing 330 ± 20 g.

Ten days before the experiments, radio-telemetric probes (TA11PA-C40, DSI, Transoma Medical, St. Paul, MN, USA) were implanted into the abdominal aorta under combined ketamine and xylazine anesthesia, along with gentamicin and followed by metamizole injections for pain relief. The arterial blood pressure (BP) signal was digitized at 1000 Hz and relayed to a computer equipped with Dataquest A.R.T. 4.0 software for analysis of cardiovascular signals.

Rats were randomized into two groups. The first group was exposed to shaker stress, with rats positioned on a platform shaking at 200 cycle/min. The second group was exposed to restraint stress, with rats placed in a Plexiglas restrainer tube (ID 5.5 cm with pores) in the supine position. Arterial blood pressure (BP) waveforms were recorded before (CONTROL) and after the first exposure to stress (SHAKER, RESTRAINT). Other phases of the experimental protocol are not relevant for this study [55].

Systolic blood pressure was derived from the arterial BP as local maxima in the BP waveforms, while the pulse interval (PI) time series was derived as the time distance between successive maximal arterial blood pressure increases. Artifacts were removed semi-automatically, first using the filter designed for cardiovascular time series [56] and then carefully visually examining the BP waveforms and residual artifacts. The signals from rats with traces of unstable health were completely excluded. A very slow varying signal component (mostly the result of rat relocation) was removed using a filter proposed by [47]. De-trended time series should be stationary at least in a wide-sense, i.e., their first and second statistical moment should be time-invariant. Then the mean value and standard deviation estimated from the time series are equal to their statistical counterparts [37], and only then the standard scaling could be reliably implemented. Thus, de-trended time series were checked using a stationarity test [57,58] and those that were not wide sense stationary were eliminated.

The final number of remaining animals per experimental group was n = 6. It was satisfactory according to the variability of the parameters in the control group rats (the statistical software “Power Sample Size Calculation”). All experimental procedures in this study were confirmed by the European Communities Council directive of 24 November 1986 (86/609/ECC), and the School of Medicine, University of Belgrade, Guidelines on Animal Experimentation.

## 3. Results and Discussion

This section presents the results of our study. Each set of the results is accompanied with the corresponding discussion.

### 3.1. Threshold Choice

**Figure 8:**
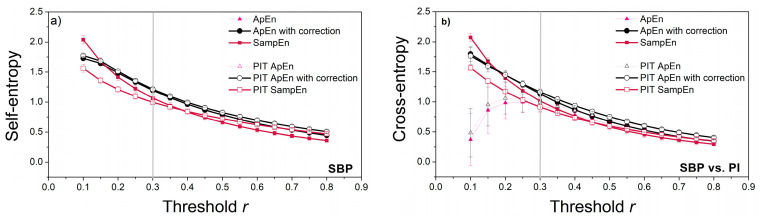
Threshold profile for signals recorded from laboratory rats: (**a**) entropy estimates of SBP; (**b**) cross-entropy estimates for SBP vs. PI signals. The estimates become stable for threshold *r* = 0.3 (vertical line).

As already pointed out, the threshold value is crucial for the consistency of entropy estimates, and its proper choice should be the first task. However, entropy is also a function of the time series length *N*—shorter time series require a larger threshold, and the relationship is not linear. A thorough analysis in [22] showed that a reliable estimation of probabilities (4) is a key factor for stable entropy measures. This requires that the threshold values be higher than the generally accepted ones. One of the methods is to plot a threshold profile, i.e., to estimate the entropy for different threshold values and fixed *N*. Figure 8 presents the *XEn* profile estimated from the cardiovascular signals, as cross-entropy requires higher threshold values than self-entropy [14,22]. Besides, real signals are a better choice for threshold profiling than stationary artificial data. The vertical lines in Figure 8 show the threshold for which the entropy estimates become consistent. This threshold value is equal to *r* = 0.3 and is adopted for further entropy estimation. The threshold evaluation method proposed in [22] gives higher threshold values, but in present study, we did not want to differ too much from the classical values.

### 3.2. Entropy Estimated from Artificial Data

The purpose of artificial data, generated in controlled conditions, is to present the reference regarding the probability integral transformation entropy estimates.

**Figure 9:**
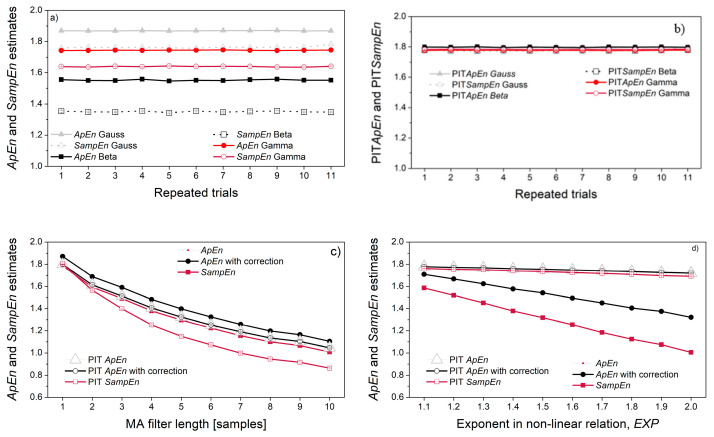
Self-entropy estimates: (**a**) Gaussian, beta and gamma distributions; (**b**) PI-transformed Gaussian, beta and gamma distributions; (**c**) MA filtered Gaussian signal; (**d**) Gaussian signal with induced non-linear transform, ***Y*** = *a*·***X****^EXP^*+ *b*.

Self-entropy estimates (*ApEn* and *SampEn*) are presented in Figure 9. As expected, the entropy of pseudo-random signals depends on their distribution (Figure 9a), but PI-transform eliminates this dependency (Figure 9b). The chosen parameters (*N* = 3000, *r* = 0.3) are sufficient to ensure reliable entropy estimates. Obviously, no correction is needed as the original and corrected *ApEn* estimates perfectly overlaps.

The dependency of signal samples, induced by the MA filter, causes an entropy decrease. The decrease is indeed due to the sample correlation, as the signal distribution remains Gaussian and decrease if entropy is seen both for original signals, and the PI-transformed signals (Figure 9c). On the other hand, the non-linear transform induced by the relation ***Y*** = *a*·***X****^EXP^* + *b* also decreases the entropy, but this decrease is due to the distribution change: PI-transform converts the distribution into uniform, and the entropy of all converted signals remains stable, regardless of the exponent *EXP* (Figure 9d).

**Figure 10:**
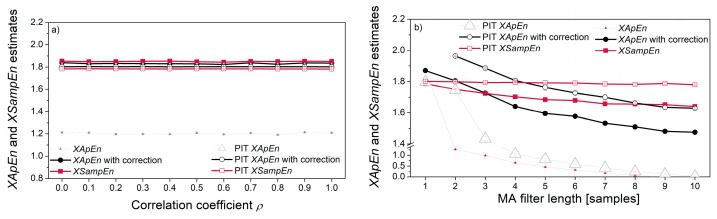

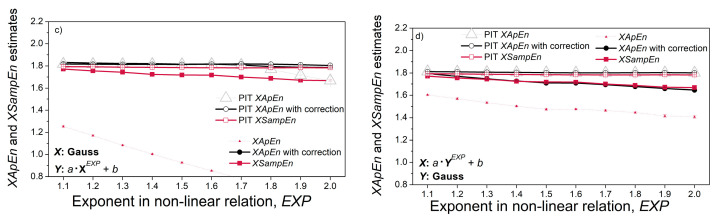
Cross-entropy estimates: (**a**) signal with beta distribution vs. signal with gamma distribution for different correlation coefficients; (**b**) signal with Gaussian distribution vs. the same signal filtered with the MA filter; (**c**) signal with Gaussian distribution (*X*) vs. its non-linearly transformed counterpart ***Y*** = *a **X**^EXP^* + *b*; (**d**) signals from (**c**) in different order, ***Y*** vs. ***X***.

Figure 10 shows cross-entropy estimates. The correlation of the signal pairs in Figure 10a has been verified using Spearman, Pearson and Kendall tests (shown in Figure 6a). However, their *XEn* estimates are constant, revealing that the entropy does not reflect the statistical correlation between two pseudo-random time series. It is in accordance with the entropy procedure, where a template vector is compared with each one of its followers, while the dependency exists only with the followers in its vicinity.

When correlation is induced by the MA filter (Figure 10b), both *XApEn* and PIT-*XApEn* decrease with the increase of filter length. *SampEn* decreases as well, but at a lower rate, while PIT-*SampEn* remains stable. *SampEn* is known to be a stable measure, due to the logarithm taken from the average matching probabilities [7]. However, this stability reduces the ability to recognize the subtle changes, and equalizing the distribution further reduces the possibility of recognition, so it might be a disadvantage.

The non-linear relationship between the signals is induced by the relation ***Y*** = *a*·***X****^EXP^* + *b*. The corresponding cross-entropies are independent of the level of exponent *EXP*, if the reference signal is a pseudo-random signal ***X*** (source signal). If the reference signal is signal ***Y***, obtained by a non-linear transform of signal ***X***, the cross-entropy decreases for *XApEn* and *XSampEn*, but the PIT counterparts remain constant. The reason is the same as for the Figure 9d: the decrease is due to the distribution of reference signal that changes as a consequence of non-linear transform; had the decrease been due to the induced non-linear coupling, the PIT entropy would have changed as well; since it has not changed, the non-linear coupling is not responsible for entropy decrease.

**Figure 11:**
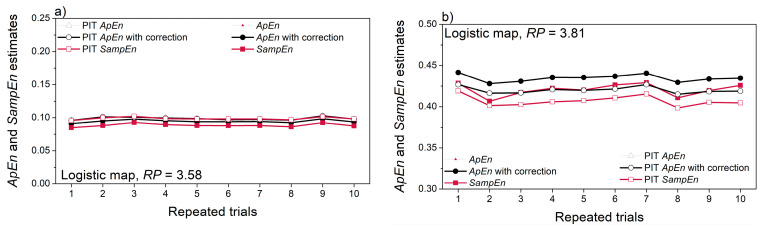

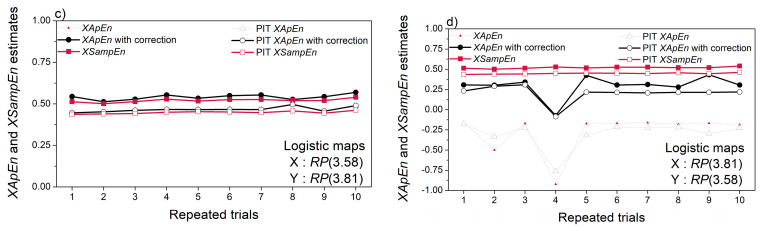
Entropy and cross-entropy estimates of deterministic chaos, generated by logistic maps: (**a**) entropy of signal ***X*** with parameter *RP* = 3.58, non-continual signal; (**b**) entropy of signal ***Y*** with parameter *RP* = 3.81; (**c**) cross-entropy ***X*** vs. ***Y***; (**d**) cross-entropy ***Y*** vs. ***X***.

The artificial time series have so far been pseudo-random signals with a given distribution. The deterministic chaos exhibits unpredictability, but not randomness. Figure 11 presents the entropy estimated from the logistic map signals. The entropy of the non-continual chaotic signal (Figure 11a) is very low, revealing a low level of its uncertainty. The second signal (Figure 11b) is genuinely chaotic and it depends on the initial conditions. For this reason, the estimated entropy values are not constant, but their changes are not significant (Figure 11b).

The absolute values of cross-entropy estimates are similar to the self-entropy in Figure 11b. This means that less predictable signal (with higher entropy) is dominant in cross-entropy estimates. On the other hand, the consistency of repeated entropy estimation governed by the reference signal: consistency of X vs. Y cross-entropy is similar to the consistency of ***X*** self-entropy (Figure 11a,c); variability of Y vs. X cross-entropy is proportional to the variability of Y self-entropy (Figure 11b,d).

### 3.3. Entropy Estimated from Cardiovascular Signals of Laboratory Rats Exposed to Stress

The parameters recorded from the laboratory rats are presented in Table 1. It reveals a significant decrease in pulse interval (increase in heart rate) in rats exposed to the restraint stress, other changes are slight and not significant.

**Figure 12:**
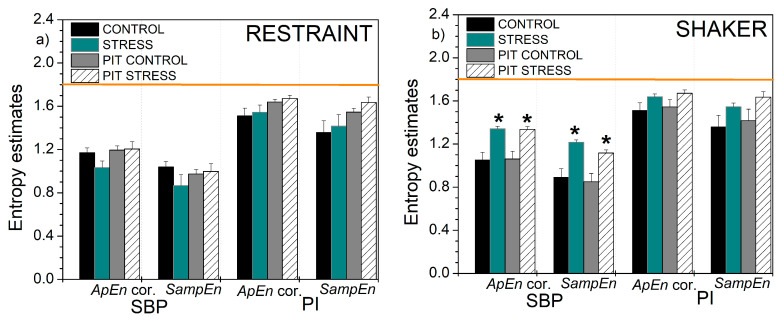

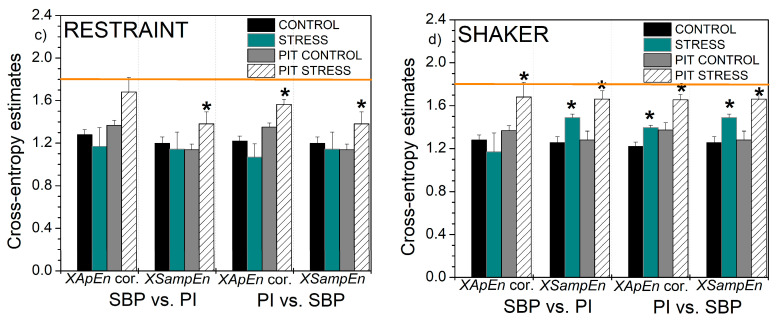
Self-entropy and cross-entropy estimates based on *ApEn* with corrections and *SampEn*: (**a**) self-entropy, rats submitted to the restraint stress; (**b**) self-entropy, rats submitted to the shaker stress; (**c**) cross-entropy, rats submitted to the restraint stress; (**d**) cross-entropy, rats submitted to the shaker stress. Results are presented as mean ± SE (standard error of mean); * denotes the difference between the control and stressed signals at the significance level of *p* < 0.05. The horizontal line shows the theoretical value for perfect random signals with uniform distribution.

The entropy estimates are given in Figure 12.

The horizontal line in Figure 12 shows the theoretical value of *ApEn*, *SampEn*, and *XEn* that can be evaluated for the random signals with uniform distribution:

(13) $$ApEn\left(unif,r\right)=SampEn\left(unif,r\right)=XEn\left(unif,r\right)=-\ln\left(\frac{4\cdot \sqrt{3}\cdot r-r^{2}}{12}\right)$$

A detailed evaluation of the theoretical value is shown in Appendix A. This is a result of perfect randomness and uniformity that can serve as a reference value, without the need to run the tedious simulation studies, e.g., surrogate data tests [51].

Considering the experimental results, the entropy of PI-transformed signals captured slightly more statistically significant differences between the cardiovascular parameters of the animals before and after exposure to stress: while classical entropies found the differences in shaker stress—SBP and PI vs. SBP, and *XSampEn* found a difference in SBP vs. PI, PIT entropy found additional differences in *XApEn* of SBP vs. PI, and, in restraint stress, in PI vs. SBP and *XSampEn* in SBP vs. PI.

Contrary to the artificial signals with the controlled outcome, the reliability of the entropy estimated from real data sources is always a subject of discussion. As already stated, a failure in estimating the matching probabilities, Equation (4), leads to an inconsistent entropy estimation. The reliability of probabilities can be checked using the Jeruchim criterion that defines the minimal signal length required to achieve ${\hat{p}}_{i}^{\left(m\right)}\left(r\right)$ within a 95% confidence interval [59]. A comprehensive theoretical analysis confirmed the traditional engineering rule that the signal length required for a reliable estimation of a binary event probability should be at least $10/{\hat{p}}_{i}^{\left(m\right)}\left(r\right)$ [59].

The ultimate case of unreliability is the matching probability equal to zero, ${\hat{p}}_{i}^{\left(m\right)}\left(r\right)=0$. This occurs if the signal length N is too short, or if the threshold r is inadequate, or if the vector length m is too long [22]. However, zero probability can occur in *XEn* for a completely logical reason: the template vector can comprise amplitudes that can never be found in another signal, so no follower vectors exists. In this case, the zero probability is not a result of an incorrect estimation, but a valid relationship between the two signals.

**Figure 13:**
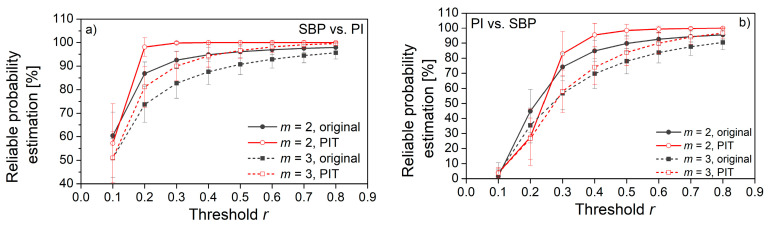
The percentage of probabilities ${\hat{p}}_{i}^{m}\left(r\right)$ that are estimated with high reliability. (**a**) SBP is the reference signal, PI is the follower; (**b**) PI is the reference signal, SBP is the follower.

**Figure 14:**
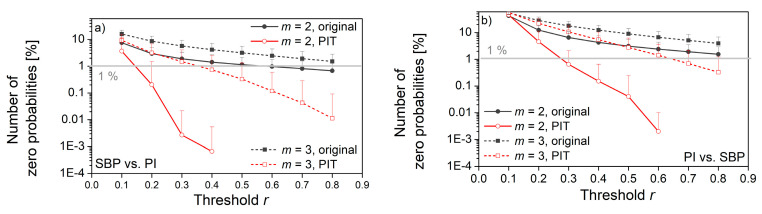
The percentage of ${\hat{p}}_{i}^{m}(r)=0$ cases that violate the entropy estimation; (**a**) SBP is the reference signal, PI is the follower; (**b**) PI is the reference signal, SBP is the follower. r. Horizontal line shows the border for which the percentage of zero probabilities is below 1%.

Figure 13 shows the percentage of reliably estimated matching probabilities, while Figure 14 shows the estimated percentage of zero probabilities.

From both figures, it can be seen that PIT signals have better performances than source signals. The increased number of reliably estimated probabilities in Figure 13 is an outcome of the uniform distribution. The signal amplitudes are equally probable, so the probability that a template finds a matching follower is increased. In distributions with exhibited tails (source signals), some of the templates are less likely to find a matching follower.

The decreased number of zero-matching probability (Figure 14) is another benefit of the probability integral transform. As already said, the distributions of signal pairs for cross-entropy can have non-overlapping segments, so some of the templates will never find the followers. After the PI-transform, the signals would be mapped into the same [0, 1] segment, and non-overlapping segments would not exist.

Figure 13 and Figure 14 also reveal the empirically obtained threshold *r* = 0.3, although slightly exceeding the traditional values from the literature (0.15–0.25), might not be sufficient for *XEn* as the values of *XApEn* and *XApEn* with correction differ. It is in accordance with the theoretical findings from [22], but we preferred to use the values that are more aligned with the traditional ones.

## 4. Conclusions

The aim of this paper was to apply the *ApEn*-based entropies and cross-entropies to the signals submitted to the probability integral transformation. PIT yields the signal with uniform distribution, keeping the signal fluctuations intact. The idea was to eliminate the influence of amplitude distribution, and to estimate the entropy where each amplitude has equal opportunity. Then the true unpredictability of the signal could be estimated without the bias induced by amplitude distribution.

The artificial environment revealed that PIT self-entropy estimates are insensitive to the linear or non-linear signal transformation, if the transformation is induced sample by sample (relationship ***Y*** = *a*·***X****^EXP^* + *b*). However, entropy estimates are sensitive to transformations that induce the dependency along the signal itself, e.g., using the MA filter. Considering the cross-entropy, its estimates remain constant when correlation coefficient between the signals ***X*** and ***Y*** increase from 0 to 1, with a conclusion that statistical correlation cannot be measured by the means of cross-entropy. Cross-entropy, on the other hand, notices if one of the signal is formed from another by inducing the correlation between its successive samples.

The chaotic signals are generated using the formula for deterministic chaos. “Chaos” did not deceive the entropy procedure, so the entropy estimates were quite low, showing the high level of signal predictability. Regardless of apparent chaotic signal appearance, the “deterministic” component could not escape the unbiased entropy measure.

Estimates of the real signals showed that PIT results of signals in stress reveal a slightly increased statistical significance than classical entropy measures. However, the main outcome is the increased estimation reliability, compared to the classical measures. The increased reliability is a consequence of the uniform amplitude distribution over [0, 1] segment and reduced number of zero-matching probabilities.

The entropy estimates of PI-transformed signals are unbiased regarding the amplitude distribution. Their reliability has improved, and a referent value—a ground truth to which entropy estimates can be compared—can be obtained by formula and not by a simulation study.

The future work will be devoted to the evaluation of errors in entropy estimation for *ApEn, SampEn, XEn*, and their PIT pairs, and to developing the methods for error attenuation. The future work will also include the continuation on thresholds role in inconsistency of entropy estimation.

## Figures and Tables

**Table 1:** Systolic blood pressure and pulse interval of rats in baseline and stressed condition.

	SBP [mmHg]		PI [ms]	
STRESS	BASELINE	STRESS	BASELINE	STRESS
SHAKER	116.28 ± 6.82	125.55 ± 8.89	167.8 ± 13.95	157.97 ± 10.98
RESTRAINT	107.08 ± 7.83	114.37 ± 24.92	179.29 ± 13.66	131.46 ± 5.71 *

The results are presented as mean ± standard deviation. * denotes the difference between the baseline and stressed signals at the significance level of *p* < 0.05.

## Appendix A

*ApEn*, *SampEn* and *XEn* of PIT transformed signals have an explicit theoretical value ground truth to which they can be compared.

*XEn* requires the signals with zero mean and standard deviation so the PIT signals have to be z-normalized (standard scale, i.e., zero mean value and unit standard deviation). The uniform probability density function of signals *x* and *y* is defined as:

(A1) $$f_{x}\left(x\right)=f_{y}\left(y\right)=\{\begin{array}{c} \frac{1}{2\cdot a},\;-a\leq x,y\leq a\\ 0,\;\mathrm{elsewhere} \end{array};\;a=\sqrt{3}.$$

The probability that two signal samples are “similar”, i.e., that their absolute difference is below the threshold *r* is equal to:

(A2) $$p=Pr\left\{\left|x-y\right|\leq r\right\}=\int_{-a}^{a}f_{x}\left(x\right)\cdot \left(\int_{y-r}^{y+r}f_{y}\left(y\right)\cdot dy\right)\cdot dx=\;=\frac{1}{4\cdot a^{2}}\cdot \left(\int_{-a}^{-a+r}\left(r+a+x\right)\cdot dx+\int_{-a+r}^{a-r}2\cdot r\cdot dx+\int_{a-r}^{a}\left(r+a-x\right)\cdot dx\right)=\;=\frac{4\cdot a\cdot r-r^{2}}{4\cdot a^{2}}=\frac{4\cdot \sqrt{3}\cdot r-r^{2}}{12}.$$

The probability that all *m* pairs of samples are similar (matching probability, Equation (4)) is equal to:

(A3) $${\hat{p}}_{i}^{m}\left(r\right)=p^{m}={\left(\frac{4\cdot \sqrt{3}\cdot r-r^{2}}{12}\right)}^{m}.$$

The summand $\Phi^{\left(m\right)}$ for (*X*)*ApEn* then becomes:

(A4) $$\Phi^{\left(m\right)}\left(r,N\right)=\frac{1}{N-m+1}\cdot \overset{N-m+1}{\underset{i=1}{\sum }}\ln({\hat{p}}_{i}^{\left(m\right)}\left(r\right))=\frac{1}{N-m+1}\cdot \overset{N-m+1}{\underset{i=1}{\sum }}\ln(p^{m})\;=\frac{N-m+1}{N-m+1}\cdot m\cdot \ln\left(p\right)=m\cdot \ln\left(p\right)$$

Similarly, $\Phi^{\left(m+1\right)}\left(r,N\right)$ = (m+1)·ln(p), so, for the case of random time series with uniform distribution, entropy estimates are equal to:

(A5) $$ApEn=XApEn=\Phi_{}^{\left(m\right)}\left(r,N\right)-\;\Phi_{}^{\left(m+1\right)}\left(r,N\right)=m\cdot \ln\left(p\right)-\left(m+1\right)\cdot \ln\left(p\right)\;=-\ln\left(p\right)$$

where *p* is given with (A2).

The results for (*X*) *SampEn* are the same:

(A6) $$SampEn=XSampEn=\Psi_{}^{\left(m\right)}\left(r,N\right)-\Psi_{}^{\left(m+1\right)}\left(r,N\right)=\ln\left(\frac{\sum_{i=1}^{N-m}{\hat{p}}_{i}^{\left(m\right)}\left(r\right)}{\sum_{i=1}^{N-m}{\hat{p}}_{i}^{\left(m+1\right)}\left(r\right)}\right)\;=\ln\left(\frac{\left(N-m\right)\cdot p^{m}}{\left(N-m\right)\cdot p^{m+1}}\right)=-\ln\left(p\right).$$
